# Listening effort in children and adults in classroom noise

**DOI:** 10.1038/s41598-024-76932-7

**Published:** 2024-10-24

**Authors:** Julia Seitz, Karin Loh, Janina Fels

**Affiliations:** Institute for Hearing Technology and Acoustics, RWTH Aachen University, 52062 Aachen, Germany

**Keywords:** Listening effort, Dual-task paradigm, Auditory cognition, Hearing in children, Working memory, Language, Auditory system

## Abstract

It is well known that hearing in noisy situations is more challenging than in quiet environments. This holds true for adults and especially for children. This study employed a child-appropriate dual-task paradigm to investigate listening effort in children aged six to ten years and young adults. The primary task involved word recognition, while the secondary task evaluated digit recall. Additionally, subjective perception of listening effort was assessed using a child-appropriate questionnaire. This study incorporated plausible sound reproduction and examined classroom scenarios including multi-talker babble noise with two signal-to-noise ratios (0 dB and −3 dB) in an anechoic and an acoustically simulated classroom environment. Forty-four primary school children aged six to ten (17 first- to second-graders and 18 third- to fourth-graders) and 25 young adults participated in this study. The results revealed differences in listening effort between the noise conditions in third- to fourth-graders and supported using the dual-task paradigm for that age group. For all three age groups, a greater subjective perception of listening effort in noise was found. Furthermore, a correlation between the subjective perception of listening effort and behavioural listening effort based on the experimental results was found for third- to fourth-graders and adults.

## Introduction

Children spend a large part of their day at school, especially with the increasing availability of after-school care. All activities in school, lessons, and breaks, involve communication and listening to peers or teachers. However, communication in schools is often disturbed by noise^1^. It is already known that noise in schools affects children’s health, development, and school performance^1^. Additionally, research has shown that children are much more impaired by noise during speech perception than adults which can affect their ability to process spoken information effectively^2,3^. This heightened susceptibility to noise raises the issue of increased processing resources being demanded of children in a noisy environment. These processing resources can be interpreted as effort, leading to the following question: How effortful is listening for children at school?

Gagné et al.^4^ defined listening effort as the amount of processing resources (perceptual, attentional, cognitive, etc.) allocated to a specific auditory task, when the task demands are high and when the listener strives to reach a high level of performance on the listening task. Currently, there are three approaches to measure listening effort^4^: subjective (e.g. by questionnaires, see^5^), psychophysiological methods (e.g., by electroencephalography (EEG)^6^), and behavioural methods (e.g., by listening experiments^7,8^).

Two behavioural methods are well-established in the literature for measuring listening effort: single-task and dual-task paradigms^9^. Single-task paradigms often utilize speech recognition tasks assessing accuracy and response times for different noise scenarios. As demonstrated in studies by Gustafson et al.^8^ with children aged seven to twelve, Prodi and Visentin^10^ with children aged eight to ten, and Prodi et al.^11^ with children aged five to seven, these response times are then used to predict listening effort for the different noise scenarios. Dual-task paradigms to measure listening effort use a speech recognition task as primary task and different types of secondary tasks. For example, Hicks and Tharpe^12^ used a reaction task for five- to eleven-year-olds and Choi et al.^13^ used a digit recall for children aged seven to fourteen. The dual-task paradigm is based on the theory of limited cognitive resources by Kahneman^14^ stating that only limited resources are available for additional tasks if increased effort is spent on listening. Kahneman’s theory was further developed into the framework of ease of language understanding (ELU)^15^ and later refined into the Framework for Understanding Effortful Listening (FUEL)^16^.

To shed light on which behavioural method to use, McGarrigle et al.^17^ compared whether dual-task paradigms including visual response times (measured in a visual monitoring task as secondary task) or single task paradigms including verbal response times (measured in a word recognition task) were more sensitive to detect differences in listening effort for children aged six to thirteen years. They suggested that single task verbal response times were more sensitive to the effects of background noise than dual-task visual response times. This aligns with the recommendations summarised in the literature review by Francis and Love^9^ who stated that the secondary tasks should draw equally from the same pool of cognitive resources as the primary task in a dual-task paradigm measuring listening effort.

Since a major influence on listening effort is the type and level of background noise, several dual-task approaches resulted in ambiguous results. Hicks and Tharpe^12^ were among the first to investigate listening effort in children (5- to 11-year-olds) using a dual-task paradigm with babble speech as background noise. However, there were no differences in listening effort among normal-hearing children for the different noise levels. They assumed, that this was because of the high signal-to-noise ratios (SNRs) applied ($\geqslant$10 dB)^12^. While Hicks and Tharpe^12^ used a reaction task as the secondary task for the dual-task paradigm, Stelmachowicz et al.^18^ and Choi et al.^13^ established a recall of five digits as the secondary task of the dual-task paradigm for children aged seven to fourteen. Both studies were conducted with two noise scenarios: silence and speech-shaped noise at an SNR of 8 dB. Then, Howard et al.^19^ used this secondary task in a dual-task paradigm to test children’s listening effort in realistic classroom SNRs ($\leqslant$4 dB). They found significant differences between digit recall performance in the single task and the dual-task condition indicating that the paradigm was suitable to measure listening effort^19^. Howard et al.^19^ recommended future studies to extend the paradigm to include spatial speaker distribution, and noise considering reverberation^19^. The latter was already respected by Picou et al.^20^ who investigated the comparison between a reverberant ($T_{30} = {834}\,{\hbox {ms}}$) and non-reverberant ($T_{30} < {100}\,{\hbox {ms}}$) noise scenario. In addition to measuring the influence of reverberation on behavioural listening effort, Picou et al.^20^ investigated subjective listening effort, which was assessed by the children using questionnaires. However, they found no significant difference between these two reverberation scenarios in the results of the dual-task paradigm or in subjective listening effort.

Inspired by the findings of Howard et al.^19^, this study aimed to assess listening effort with more acoustic realism, taking into account the spatial distribution of the speakers and an acoustically typical German classroom scenario. The dual-task approach with digit recall as secondary task was chosen to investigate listening effort due to its similarity to classroom situations, where listening and learning are often combined with other tasks, e.g., taking notes. Leist et al.^21^ showed that children memorise digits verbally, which implies that the primary task (word recognition) and the secondary task (digit recall) draw from the same pool of cognitive resources^9^, thus the digit recall was considered suitable as secondary task. In addition to the dual-task paradigm, this study applied the subjective measurement approach using a questionnaire on children’s listening effort.

The dual-task paradigm assessed listening effort with a combination of two approaches, as suggested by Francis and Love^9^. First, the comparison of dual-task and single task performance in silence was statistically analysed as proposed by Gagné et al.^4^. By comparing the single secondary task performance to the dual-task performance, the specific contribution of the listening task to the overall cognitive load could be determined providing insights into the required effort. Second, listening effort was measured as the comparison of secondary task performance under different noise conditions^22^. This method revealed an assessment of how different plausible acoustic classroom noise scenarios impacted cognitive resources in order to identify any possible differences in listening effort.

As recent research measuring listening effort with dual-task paradigms in children mostly investigated large age groups spanning several years (e.g., six^12^ or eight years^7^), it was decided to investigate two smaller age groups based on German primary school classes to respect children’s development better. One age group included first- to second-graders (aged six to seven years), and the other age group included third- to fourth-graders (aged eight to ten years). Additionally, young adults were investigated as an age group because Picou et al.^7^ found differences in dual-task performance between children (aged nine to seventeen) and young adults.

Four hypotheses were formulated and derived from the existing literature for this study. First, it was hypothesised that listening effort could be measured using the proposed dual-task approach meaning that the results by Howard et al.^19^ could be reproduced with the extended paradigm. This would be reflected in participants showing worse performance in the secondary task (digit recall) when performed in the dual-task compared to the single-task condition, signalling a higher listening effort. Second, it was assumed that the performance in the dual-task condition (measured as the secondary task’s ER and RT, and the primary task’s RT) would be affected by the different background noise scenarios, i.e. performance would be worse in any noise scenario compared to quiet, at an SNR of −3 dB compared to an SNR of 0 dB, and with additional room effects compared to anechoic conditions. The third hypothesis was that the error rate in the word recognition task would increase with each noise condition compared to quiet, with an SNR of −3 dB compared to an SNR of 0 dB, and with additional room effects compared to anechoic conditions. Finally, it was hypothesised that a significant correlation exists between the behavioural listening effort assessed by the dual-task paradigm and the subjective listening effort assessed by questionnaires.

## Methods

### Participants

The listening experiment was conducted with 44 primary school children aged six to ten (mean age 7.5 years, 42.86% female) and 25 adults aged 21 to 37 years (mean age 26, 28% female). The children were recruited through cooperation with primary schools in Aachen, Germany, and via e-mail notifications in which the guardians had previously expressed their interest in participating in listening tests. Adults were recruited through the university. The inclusion criteria for participants were German-speaking, normal hearing (HL $\leqslant$ 20 dB for $f = [250, 500, 1000, 2000, 4000]{\hbox {Hz}}$^23^), and not diagnosed with attention deficit hyperactivity disorder (ADHD) or epilepsy. The experimental data from nine children were excluded from the data analysis: one child had to cancel during participation due to illness, another child due to technical problems, two children were excluded because they did not have normal hearing, two children due to suffering from ADHD and three children had to be excluded due to not having sufficient German knowledge (based on parent reporting). No adults needed to be excluded; however, 11 out of 25 adults participated in the prestudy testing the experimental design. Ethical approval by the Medical Ethics Committee at RWTH Aachen University was obtained prior to the recruitment of the participants (EK 23-242) and the study was performed in compliance with the Declaration of Helsinki guidelines. As compensation for their participation, children and adults received a 10€-voucher from a local book and toy store that also offers online dispatch.

### Experiment task

A dual-task paradigm was chosen as the experiment task to measure listening effort. This paradigm consisted of two tasks performed simultaneously, the primary task and the secondary task. The participants were told to concentrate primarily on the primary task. It involved word recognition using a word-to-picture matching task^3,24^. Each trial of this task contained one spoken target word to be identified and four phonologically similar response choices that were represented by child-appropriate pictures. The task consisted of three training word lists and five experiment word lists balanced across participants. The target words were recorded by a trained, native German-speaking female with a Sennheiser MD 421-II-4 Dynamic Studio Microphone in a sound-attenuated booth at a sampling rate of 44.1 kHz and 16-bit resolution. The recordings were loudness-normalized according to EBU R-128^25^ using the audio software Audacity^26^.

**Fig. 1:**
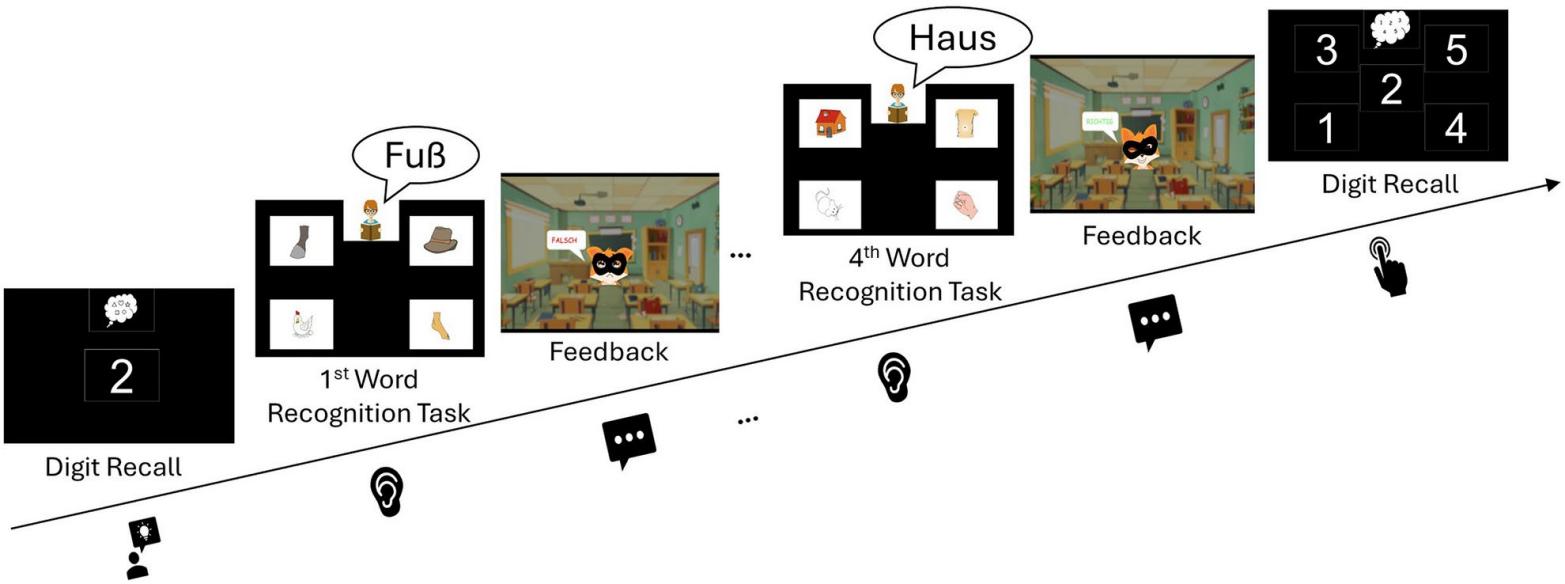
The structure of one trial unit with the digit recall (secondary task) enclosing four word recognition trials (primary task). For the word recognition trials, the German words, “Fuß” (Engl. foot) and “Haus” (Engl. house) are depicted. The children’s version of the digit recall comprising only five digits is shown here.

The secondary task was a digit recall task in which adults had to remember seven digits (from one to seven) and children five digits (from one to five). The algorithm ensured that no digit was repeated and no more than two consecutive steps, up or down, occurred (e.g., “1-2-3-4” or “7-6-5-4” would not be allowed). The experimental procedure of one trial unit (see Fig. 1) was as follows: First, the digit sequences to be remembered were shown one after another displaying each digit for 1 s. They had to be remembered throughout four word recognition trials. In each of these four word recognition trials, the participants had to listen to the spoken target word and insert their responses by clicking on the corresponding coloured drawing. Subsequently, the participants had to respond to a mixed-number array inserting the memorised digits.

### Assessing subjective listening effort

A recent literature review^27^ showed that different measures of listening effort are poorly correlated. This finding supported the multidimensional concept of listening effort meaning that different measures assess different aspects of effort on the perception pathway^27^. Thus, in this study, listening effort was measured not only behaviorally but also subjectively using a participant questionnaire. In their review paper, Francis and Love^9^ summarised that by answering the question of how effortful listening was, participants might also answer two other questions such as “How do you feel?” and “How did you perform?”. Based on their summary, three questions were chosen to categorise the effort exerted by participants in the different noise conditions in the listening experiment. The participants were asked to answer these three questions after every experiment block:

1. Wie geht es Ihnen/dir gerade in diesen Moment bei dem Hörversuch?
  Sehr gut, gut, mittelmäßig, schlecht, sehr schlecht
  *Engl.: How are you feeling at this moment during the listening test?*
  *Very good, good, average, bad, very bad*
2. Was denken Sie/ denkst du, wie gut haben Sie/ hast du die Aufgabe gelöst? Sehr gut, gut, mittelmäßig, schlecht, sehr schlecht
  *Engl.: How well do you think you solved the task?*
  *Very good, good, average, bad, very bad*
3. Wie anstrengend war es für Sie/ dich, diese Aufgabe zu bearbeiten? Gar nicht anstrengend, wenig anstrengend, mittelmäßig anstrengend, eher anstrengend, sehr anstrengend
  *Engl.: How effortful was it for you to do this task?*
  *Not at all effortful, little effortful, moderately effortful, rather effortful, very effortful*

The questions were answered on a five-point Likert scale with the answer options depicted above. The answer options for each question were coded with a number from zero (e.g., “very good”) to four (e.g., “very bad”). To create a subjective score on listening effort, the three questions’ scores were summed for each noise condition and each participant leading to continuous values between zero (no effort) and twelve (the most effort).

### Experimental procedure

**Fig. 2:**
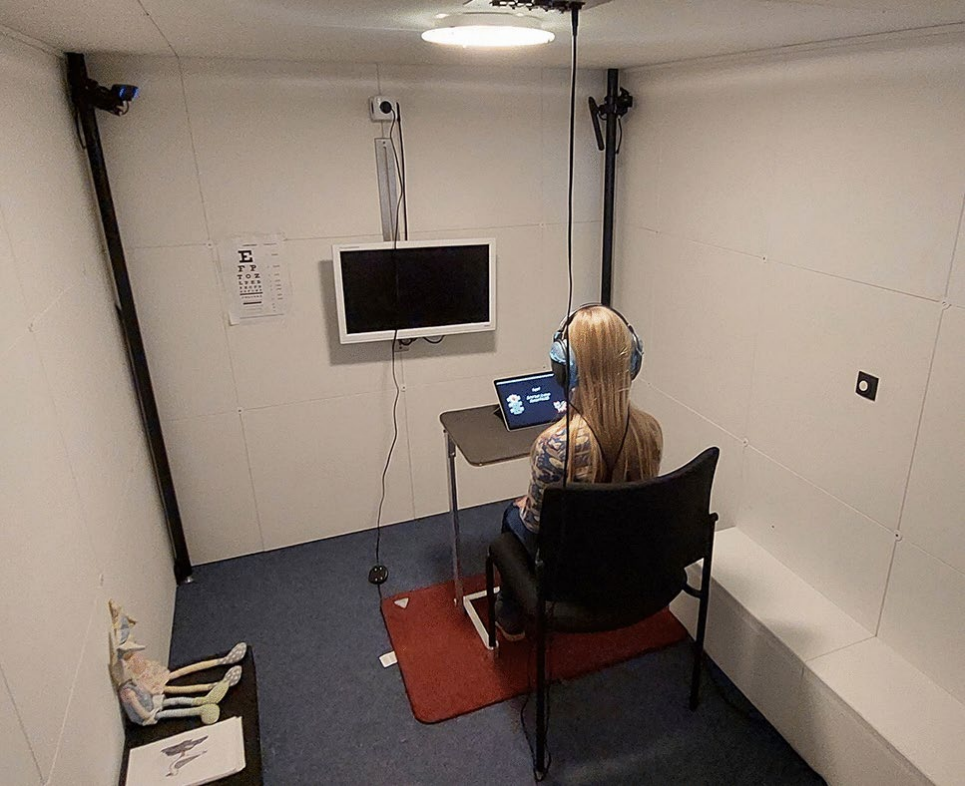
A child participant during the listening experiment.

The listening experiment with adults was conducted in April and May 2023 and the listening experiment with children took place in October and November 2023. The listening experiment was carried out in the mobile hearing laboratory, a caravan with an integrated hearing booth^28^ (see Fig.  2). With the caravan, the listening experiment could be carried out on-site in the primary school.

Before the start of the experiment, informed consent and the questionnaire on the participants’ social background were collected from the adult participants and the children’s guardians respectively. Then, the participants performed a vision test (Snellen^29^) and a pure-tone audiometry (250 Hz - 4000 Hz). Furthermore, children were asked to name selected images from the word-to-picture matching task^3,24^ to ensure task understanding. As the experiment was designed child-appropriately including gamification, the participants were introduced to a story leading them through the listening experiment. The story was about Mrs. Fox, a teacher, who has favourite words to be recognised and likes to play memory games together with the participants.

The experimental introduction comprised three parts: First, participants practised the word recognition (primary) task (32 trials), then the digit recall (secondary) task (9 trials) and finally both tasks concurrently in the form of the dual-task (4 trial units). The first two introduction parts for practising the word recognition task and the digit recall task also served as a baseline for conducting the experimental task as a single task without background noise. Thus, the first four trials of the word recognition task were taken as training and the following 28 trials were counted as the baseline. The same applies to digit recall: the first two trials were used for practising and the following seven trials formed the baseline. Having the training and the baseline at the beginning of the experiment is common practice in dual-task designs to reduce the duration of the experiment^7,13^ and it is still applied in up-to-date research^30^. The importance of the primary task (word recognition) was highlighted in the instructions and supported by feedback occurring after each word.

Following the introduction, the participants could ask comprehension questions and then the experiment’s main part started. The experiment consisted of five blocks, each containing one of the five noise conditions. Each experiment block consisted of seven trial units (7 digit recall and 28 word recognition tasks). The noise conditions were balanced across participants for each participant ID. Between each block, participants had the opportunity to take a break. The participants entered the experiment’s responses on a tablet. The error rates (ERs), which range from zero to one, and response times (RTs) in seconds were measured for both tasks. After each experimental block, participants completed the questionnaire on subjective listening effort.

### Plausible virtual sound reproduction

The listening experiment aimed to create an acoustic scenario that was as close to reality as possible. To achieve this goal, the sound reproduction was spatialised and individualised, and the speaker positions and background noises were chosen as in the classroom. The sounds were reproduced binaurally, which corresponds to the human way of hearing and enables, for example, spatially distributed sound sources. For binaural reproduction, a generic head-related transfer function of the artificial head of the Institute for Hearing Technology and Acoustics^31^ was used. This generic head-related transfer function was individualised to respect the individual differences between participants’ hearing. Therefore, at the beginning of the experiment, the head sizes (head width, depth, and height) of the participants were measured. Based on these, interaural-time difference cues were adjusted^32^. This is especially important for children due to their smaller head sizes. For headphone equalisation of the open headphones used (HD 650 by Sennheiser), the headphone-related transfer function of each participant was measured at the beginning of the experiment and respected for audio reproduction^33^.

Additionally, with regard to the speaker positions and background noise, the listening experiment aimed to create a scenario that was as close to being realistic as possible. It was a challenge to design a plausible classroom noise as there are many different noise sources in a classroom that influence children differently. In this study, the main noise source was considered to be the children themselves; hence, German children’s multi-talker babble noise (MTB) was chosen as a plausible noise signal for this listening experiment. The MTB was recorded in an anechoic room consisting of four German girls (aged eight to nine) reading a short fairy tale in a competing speech manner^34^. Virtual acoustic scenes were created using MATLAB R2022a^35^ and the MATLAB integration Virtual Acoustics 2022a^36^ using the room-acoustic renderer. The virtual spatial speaker positions were arranged to simulate a typical classroom setting of group work, with spoken target words always originating from the front at $0^{\circ }$ as if they came from a teacher. In addition, the multi-talker babble noise was played back from each of the four positions surrounding the listener: front-right ($315^{\circ }$), back-right ($225^{\circ }$), back-left ($135^{\circ }$), and front-left ($45^{\circ }$) creating the impression as if other children were babbling around the listener. All speaker positions were at a distance of 2 m from the listener. Depending on the participants, whether they were children or adults, the receiver and speaker heights were adjusted according to their sizes for children (.8 m) and adults (1.2 m).

In their outlook, Howard et al.^19^ motivated future studies to consider not only the spatial distribution of noise and speakers but also the reverberant characteristics of a classroom. Both were taken into account in this listening experiment. Two different noise scenarios were chosen: anechoic and with room effects of a typical classroom. The latter was achieved by creating a virtual acoustic scene that made the participants acoustically feel as if they were sitting in a real classroom, taking into account room effects such as reverberation. The simulated room with the respective receiver position was chosen from the EduRA Database^37^ (Cluster 1) and was based on acoustic measurements in real classrooms. The simulated classroom had a floor area of ${9.1}\,{\hbox {m}} \times {7.5}\,{\hbox {m}} = {68.25}\,{\hbox {m}}^{2}$, a volume of ${68.25}\,{\hbox {m}}^{2} \times {3.22}\,{\hbox {m}} = {219.77}\,{\hbox {m}}^{3}$, and a reverberation time $T_{30} = {0.63}\,{\hbox {s}}$ (averaged over the octave bands $f = {0.5}\,{\hbox {kHz}}, f = {1}\,{\hbox {kHz}}$ and $f = {2}\,{\hbox {kHz}}$). Mealings and Buchholz^38^ found that $T_{30} = {0.63}\,{\hbox {s}}$ is a typical reverberation time for primary school classrooms. In the simulated classroom, binaural room impulse responses for all speaker positions were simulated using RAVEN^39^. In addition to the two noise scenarios, two different SNRs, 0 dB and $-3$ dB, were chosen according to the study by Klatte et al.^2^. Before the experiment conduction, the loudness was calibrated. The sound pressure level of the word to be perceived was fixed at 60 dB. The noises were calibrated to SNRs of 0 dB and −3 dB respectively. Hence, in total, there were five different noise conditions: no noise (Q), anechoic MTB with SNRs of 0 dB (A1) and −3 dB (A2) and MTB with room effects with SNRs of 0 dB (R1) and −3 dB (R2). The abbreviations in brackets are used to present the results more clearly.

## Results

For the data analysis of the experiment and the questionnaire results, the children were grouped into two age groups according to their grades. This led to three age groups: 25 adults (mean age: 26 $\pm 3.7$ years; 28% female), 17 first- to second-graders aged six to seven years (mean age: 6.47 $\pm 0.5$ years; 58.82% female), and 18 third- to fourth-graders aged eight to ten years (mean age: 8.56 $\pm 0.7$ years; 27.78% female). The data analysis was conducted with IBM SPSS Statistics^40^ and plots were generated using the R toolbox ggplot2^41^. For all analyses, the significance level $\alpha =0.05$ was applied.

For each experimental task, word recognition and digit recall, error rates (ERs) and response times (RTs) were evaluated as dependent variables. For the word recognition task, response time was measured from the onset of word playback until response insertion and the error rate was calculated by dividing the number of incorrect responses per block by the total number of trials in that block. For digit recall, response time was measured from the moment the digits were visible until the insertion of the last digit, and the error rate was calculated per trial unit by dividing the number of incorrectly recalled digits by the total number of digits. For paradigm validation, response times and error rates of the secondary task were evaluated using two-factor repeated measures analysis of variance (ANOVA). Task type (single or dual-task) acted as a within-subjects variable, and age group was a between-subjects variable. To investigate the noise-induced differences between the tasks, four two-factor repeated-measures ANOVAs, one for each task (primary and secondary) and one for each dependent variable (ER and RT) were conducted. The different noise conditions acted as within-subjects variables and age as a between-subjects variable. The questionnaire scores were analysed with a two-factor ANOVA with noise block acting as the within-subjects variable, and age acting as the between-subjects variable.

For the ANOVAs on the experiment and the questionnaire results, normality was checked based on QQ plots, and the data were mainly normally distributed. The only noise condition that stood out was the no noise condition, which was not normally distributed. The ANOVAs were still performed because that analysis is robust against this non-normal distribution^42,43^. In addition, Mauchly’s test for sphericity was conducted before each analysis and the Greenhouse-Geisser correction was applied where the assumption of sphericity was violated.

Before the analysis, the first four word recognition trials and the first memory trial were filtered out for each noise condition. This was done to avoid participant adaptation effects so that the data would better reflect their performance without initial fluctuations. For the introduction where the latter task part was the single task baseline, the first four word recognition trials for the primary task and the first two memory trials for digit recall were filtered out. Then, the error rate was computed for the word recognition task per block. Primary or secondary task trials exceeding three times the standard deviation per participant for response time were filtered out for the response time and the error rate^44^. For the ANOVA on the questionnaire scores, no filtering was applied because only one data point per participant was collected for each noise condition.

### Paradigm validation: comparison between single and dual-task results of the secondary task

The difference between dual-task and single-task ER for digit recall showed decreasing values with age in the quiet environment: $ER_{diff} =0.296$ for first- to second-graders, $ER_{diff} =0.158$ for third- to fourth-graders, and $ER_{diff} =0.037$ for adults. A two-factor repeated-measures ANOVA demonstrated significant main effects of task type (*F*(1, 57) = 38.190, $p <0.001$, $\eta _p^2=0.401$), age (*F*(2, 57) = 37.216, $p <0.001$, $\eta _p^2=0.566$), and the interaction between task type and age (*F*(2, 57) = 8.324, $p <0.001$, $\eta _p^2=0.226$) on the error rate in the digit recall task. The post-hoc test for the interaction of age group and task type revealed statistically significant comparisons for single and dual-task for the first- to second-graders ($p <0.001$, $M_{diff}=0.296$) and the third- to fourth-graders ($p =0.002$, $M_{diff}=0.158$), whereas there was no significant difference for the adults.

**Fig. 3:**
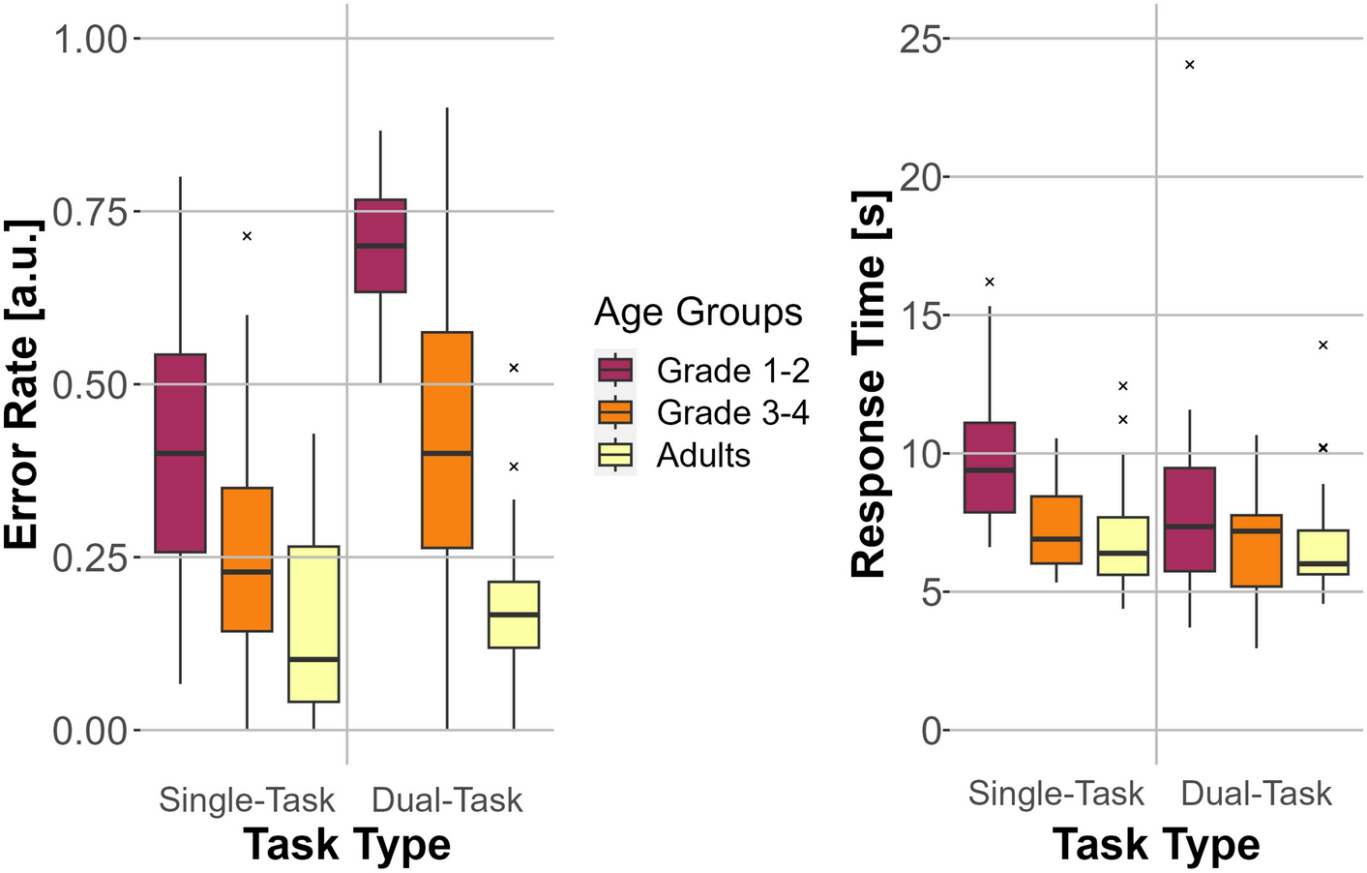
Single and dual-task comparisons for error rate (left) and response time (right) for the secondary task (digit recall) for the three age groups (adults, first- to second-graders, and third- to fourth-graders). The error rate/ response time is depicted on the *y*-axis, and the task type is shown on the *x*-axis. Box plots show the median (middle line), and interquartile range of the data distributions.

The comparison for the differences in response time between single and dual-task conditions for digit recall in quiet showed surprisingly greater response times for the single task namely, 61 ms for adults, 465 ms for third- to fourth-graders and 1733 ms for first- to second-graders. A two-factor repeated-measures ANOVA on response times for the digit recall task revealed a statistically significant effect of task type (*F*(1, 57) = 5.616, $p =0.021$, $\eta _p^2=0.090$) and grade (*F*(2, 57) = 4.485, $p =0.016$, $\eta _p^2=0.136$). The interaction between task type and grade did not show significant differences (*F*(2, 57) = 2.390, $p = 0.101$, $\eta _p^2=0.077$) Bonferroni corrected post-hoc tests revealed significantly greater response times for first- to second-graders compared to adults ($p = 0.046$, $M_{diff} = {2.02}\,{\hbox {s}}$) as well as compared to third- to fourth-graders ($p = 0.023$, $M_{diff} = {1.96}\,{\hbox {s}}$). The Bonferroni-corrected post-hoc comparison between single and dual-task was also statistically significant, with, on average, 0.76 s greater response times for the single task ($p =.021$). Both comparisons, for response time and error rate, are visualized in Fig.  3.

### Comparison between noise blocks

A two-factor repeated-measures ANOVA was conducted for the primary and secondary tasks for the error rate and response time, respectively, to investigate the noise-induced differences between the different noise conditions.

#### Secondary task—error rates

**Fig. 4:**
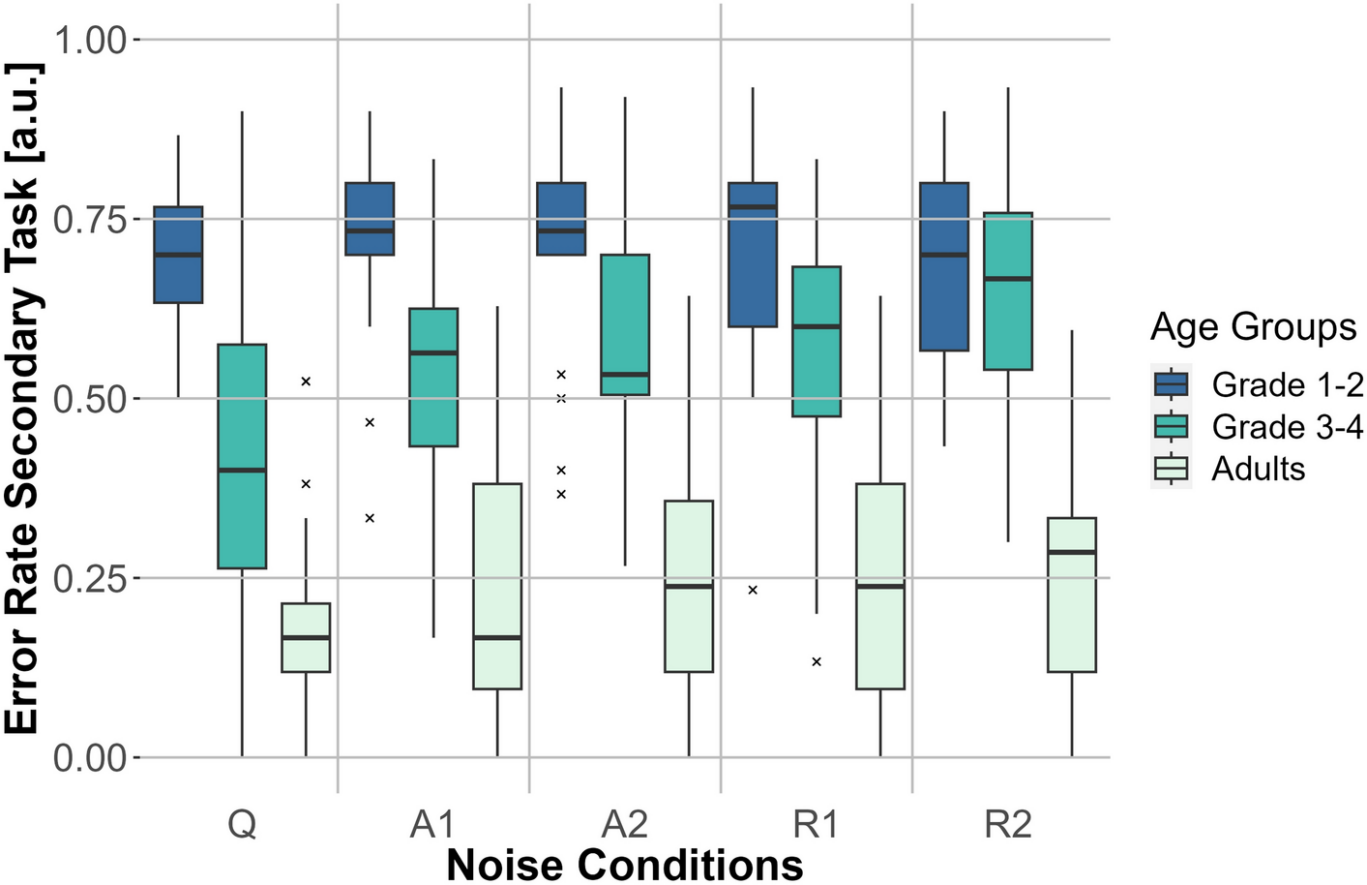
Error rate for the secondary task (digit recall) for the three age groups (adults, first- to second-graders, and third- to fourth-graders) for the five noise conditions: no noise (Q), anechoic MTB with SNRs of 0 dB (A1) and −3 dB (A2) and MTB with room effects with SNRs of 0 dB (R1) and −3 dB (R2). The error rate is depicted on the *y*-axis and the noise conditions are represented on the *x*-axis. Box plots show the median (middle line), and interquartile range of the data distributions.

A two-factor repeated-measures ANOVA demonstrated significant main effects of noise condition (*F*(4, 228) = 5.075, $p <0.001$, $\eta _p^2=0.082$), age (*F*(2, 57) = 77.918, $p <0.001$, $\eta _p^2=0.732$), and the interaction of noise condition and age (*F*(8, 228) = 2.236, $p = 0.026$, $\eta _p^2=0.073$) for the error rates of the digit recall task. Figure 4 shows the results. Post-hoc testing using Bonferroni correction indicated that for third- to fourth-graders the ER was significantly higher for the noise blocks R1 ($p =0.005$) and R2 ($p <0.001$) than they were for the Q condition. The Bonferroni-corrected post-hoc comparison of ERs between the noise conditions Q and A2 was marginally significant with $p =0.05$ for third- to fourth-graders. However, there was no significant difference in the ERs between the noise conditions of first- to second-graders ($p >0.05$) or adults ($p >0.05$).

#### Secondary task—response times rates

The two-factor repeated-measures ANOVA (Greenhouse-Geisser corrected) on digit recall response times did not reveal any significant main effect for the noise condition (*F*(3.047, 173.689) = 0.121, $p = 0.949$, $\eta _p^2=0.002$), age group (*F*(2,57) = 0.686, $p = 0.508$, $\eta _p^2=0.024$), or the interaction between age group and noise condition (*F*(6.094, 173.689) = 0.950, $p =0.462$, $\eta _p^2=0.032$).

**Fig. 5:**
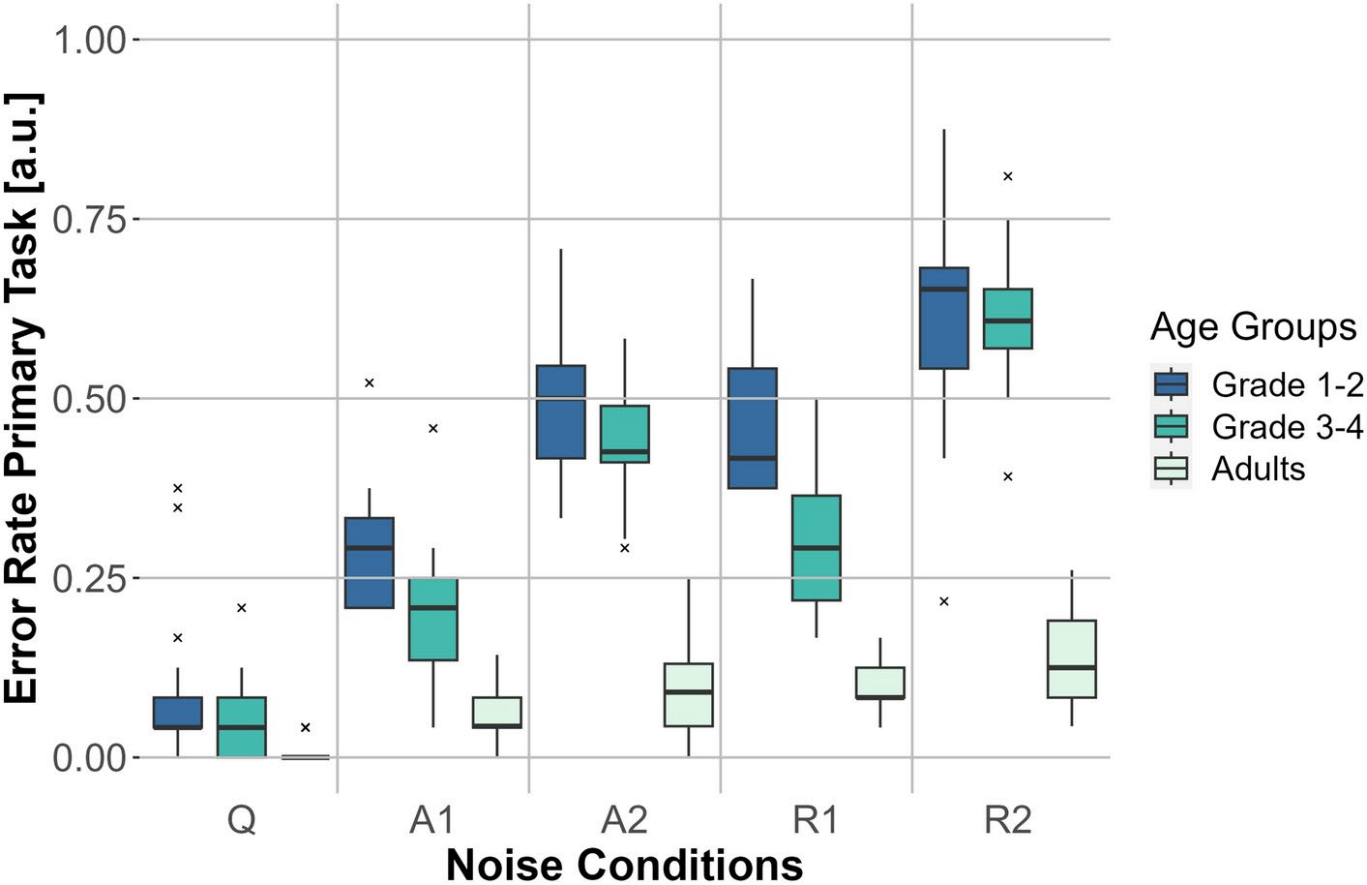
Error rate for the primary task (word recognition) for the three age groups (adults, first- to second-graders, and third- to fourth-graders) for the five noise conditions: no noise (Q), anechoic MTB with SNRs of 0 dB (A1) and −3 dB (A2) and MTB with room effects with SNRs of 0 dB (R1) and −3 dB (R2). The error rate is represented on the *y*-axis and the noise conditions are depicted on the *x*-axis. Box plots show the median (middle line), and interquartile range of the data distributions.

**Fig. 6:**
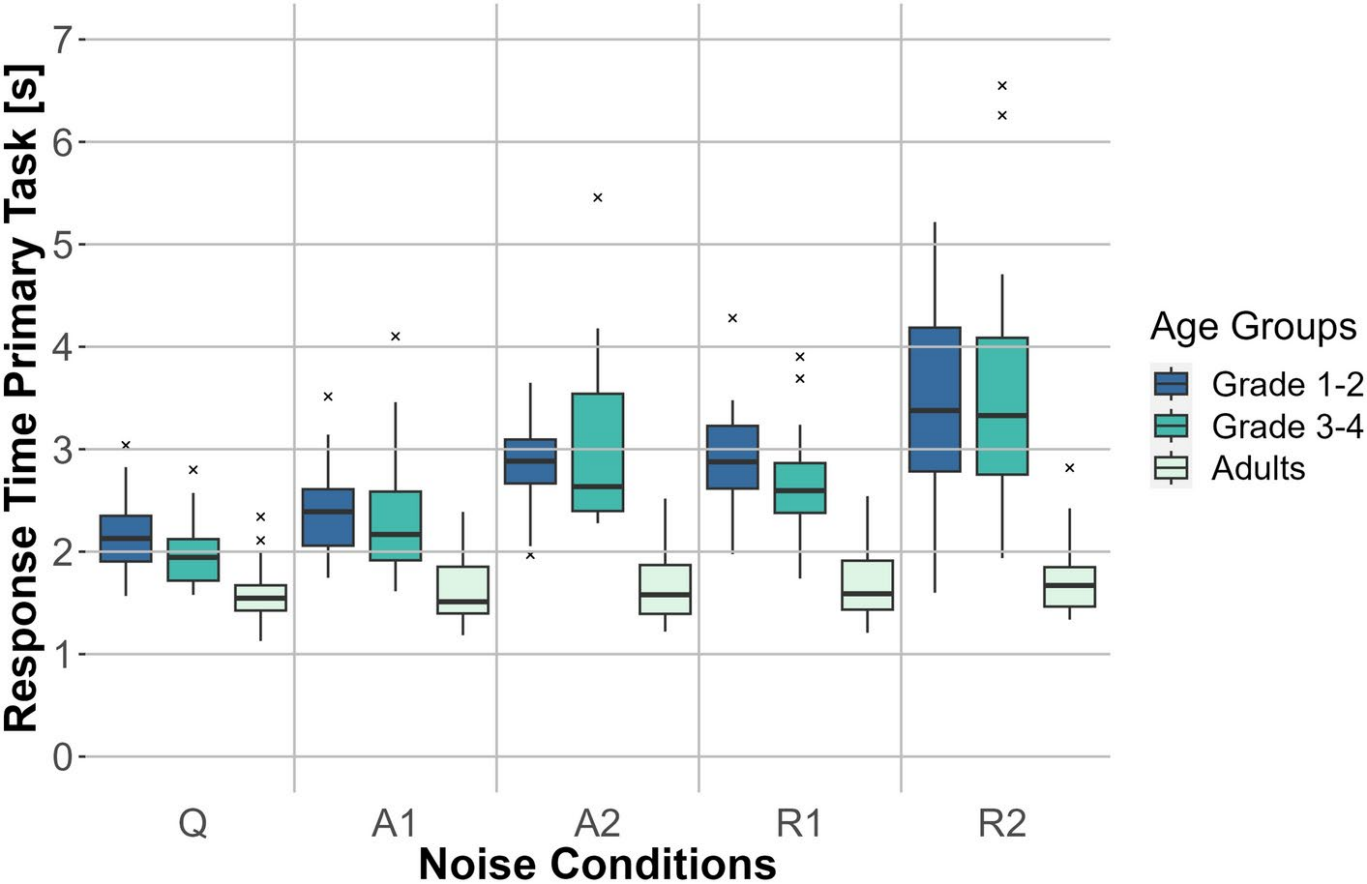
Response time for the primary task for the three age groups (adults, first- to second-graders, and third- to fourth-graders) for the five noise conditions: no noise (Q), anechoic MTB with SNRs of 0 dB (A1) and −3 dB (A2) and MTB with room effects with SNRs of 0 dB (R1) and −3 dB (R2). The response time is depicted on the *y*-axis and the noise conditions are depicted on the *x*-axis. Box plots show the median (middle line), and interquartile range of the data distributions.

#### Primary task—error rates

A two-factor repeated-measures ANOVA (Greenhouse-Geisser corrected) to compare error rates in the word recognition task showed significant differences for the main effect of age group (*F*(2, 57) = 251.931, $p <0.001$, $\eta _p^2=0.898$), noise condition (*F*(3.333, 189.961) = 235.923, $p <0.001$, $\eta _p^2=0.805$), and the interaction of the two (*F*(6.665, 189.961) = 35.132, $p <0.001$, $\eta _p^2=.552$) (see Fig. 5).

Bonferroni-corrected post-hoc tests for the interaction of age group and noise condition were significant for all comparisons between blocks for first- to second-graders ($p\leqslant 0.001$) and for third- to fourth-graders ($p\leqslant 0.001$). Additionally, the comparisons between blocks for adults were significant for the comparison of the blocks Q and A2 ($p <0.001$), Q compared to R1 ($p < 0.001$), Q compared to R2 ($p <0.001$), and marginally significant for the comparison A1 compared to R2 ($p =0.005$). Looking at the comparison of first- to second-graders and third- to fourth-graders for specific noise blocks, there were significant differences between these two age groups for blocks A1 ($p =0.003$) and R1 ($p < 0.001$).

#### Primary task—response times

A two-factor repeated-measures ANOVA (Greenhouse-Geisser corrected) comparing response times in the word recognition task showed significant results for the main effects of age group (*F*(2, 57) = 38.098, $p <0.001$, $\eta _p^2=0.572$), noise condition (*F*(2.509,143.011) = 59.037, $p <0.001$, $\eta _p^2=0.509$), and the interaction of noise condition and age group (*F*(5.018,143.011) = 12.887, $p <0.001$, $\eta _p^2=0.311$), The Bonferroni-corrected post-hoc tests for the interaction between noise condition and age group showed no significant difference between the noise conditions for adults ($p >0.05$). However, all comparisons of noise conditions were significant for third- to fourth-graders ($p \leqslant 0.019$). Additionally, for the group of first- to second-graders, all comparisons of noise conditions were significant ($p \leqslant 0.033$) except for the comparison between A2 and R1 ($p >0.05$). The interactions are plotted in Fig. 6.

### Validation of the child-appropriate questionnaire on subjective listening effort

The questionnaire on subjective listening effort consisted of three items: questions on well-being, task performance and task effort. Cronbach’s alpha^45^ was used to test the internal consistency of the questionnaire. Cronbach’s alpha for the three items was $\alpha =0.653$ for adults, $\alpha =0.623$ for first- to second-graders, $\alpha =0.626$ for third- to fourth-graders, and $\alpha =0.607$ for all age groups combined.

### Subjective listening effort ANOVA

**Fig. 7:**
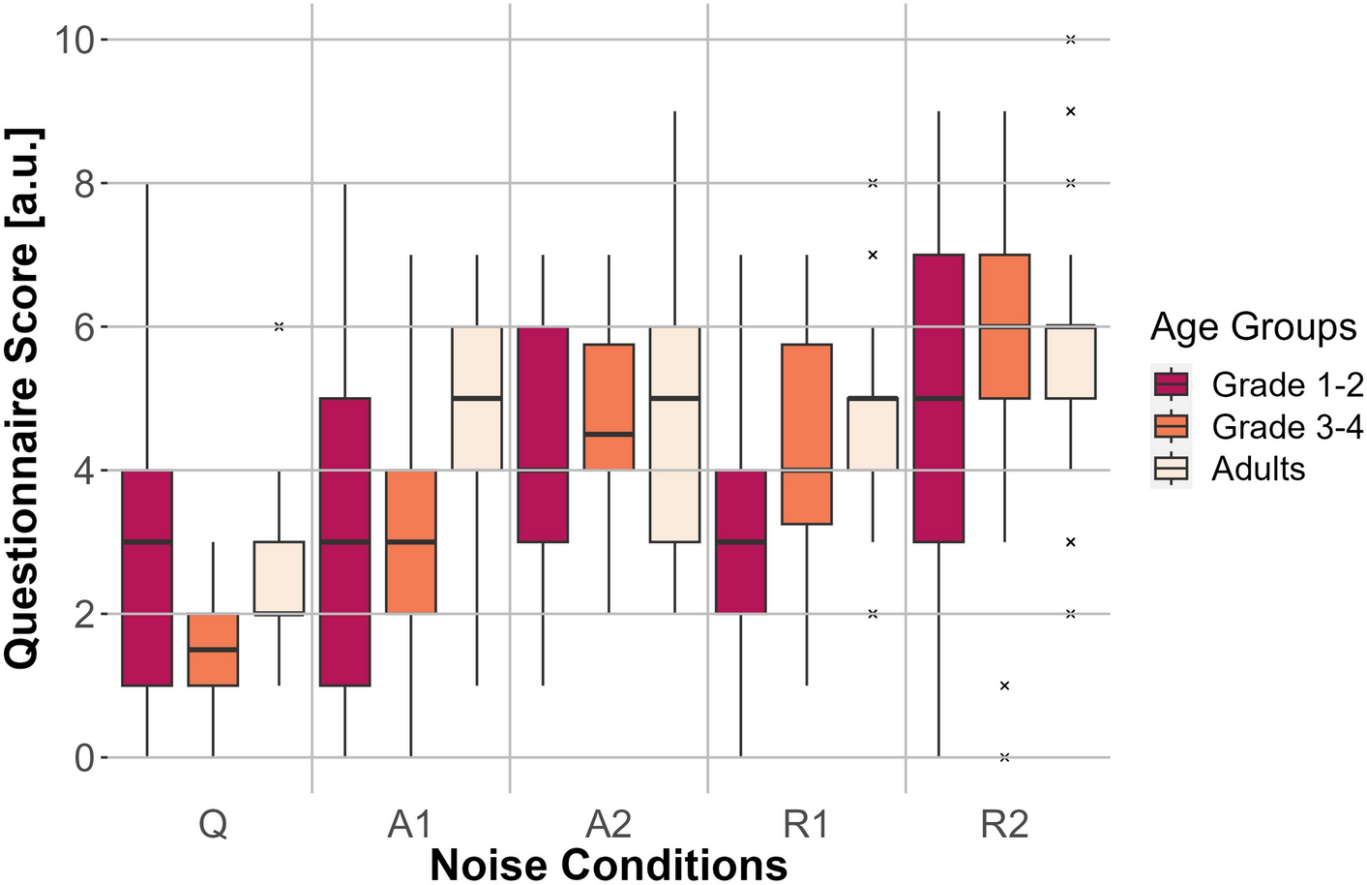
Questionnaire score for the three age groups (adults, first- to second-graders, and third- to fourth-graders) for the five noise conditions: no noise (Q), anechoic MTB with SNRs of 0 dB (A1) and −3 dB (A2) and MTB with room effects with SNRs of 0 dB (R1) and −3 dB (R2). The questionnaire score is depicted on the *y*-axis and the noise conditions are shown on the *x*-axis. Box plots show the median (middle line), and interquartile range of the data distributions.

The two-factor ANOVA (Greenhouse-Geisser corrected) comparing subjective listening effort scores revealed significant differences for the main effect of noise condition (*F*(3.426, 195.260) = 30.006, $p <0.001$, $\eta _p^2=0.345$) and the interaction between noise condition and age group (*F*(6.851, 195.260) = 3.041, $p = 0.005$, $\eta _p^2=0.096$). The main effect of age group (*F*(2, 57) = 2.241, $p > 0.05$, $\eta _p^2=0.116$) was not significant. The results are depicted in Fig. 7.

The Bonferroni-corrected post-hoc analyses of the interaction between noise condition and age group revealed that the first- to second-graders had higher scores for the subjective estimation of listening effort than did the third- to fourth-graders for the Q condition ($p =0.011$). However, first- to second-graders had significantly lower values than adults for the R2 condition ($p = 0.004$). Considering the results of the post-hoc tests with Bonferroni correction for the interaction between noise condition and age group from the perspective of the age group, it was found that

- for adults, all noise conditions differed significantly from the Q condition ($p <0.001$)
- for first- to second-graders,
  - Q differed significantly from R2 ($p =0.018$)
  - A2 differed significantly from R1 ($p =0.022$)
  - A1 differed significantly from R2 ($p =0.033$)
  - R1 differed significantly from R2 ($p =0.008$)
- for third- to fourth-graders, the Q condition differed significantly from all other noise conditions ($p \leqslant 0.001$) and the A1 condition differed significantly from the R2 condition ($p <0.001$).

### Correlation between behavioural and subjective listening effort

To check for any correlation between the overall score on subjective listening effort and the behavioural results of the listening experiment, a Spearman correlation $r_s$ was tested between the results of the ERs and the RTs of the primary and secondary task and the corresponding overall questionnaire score. This means that for each participant five data points, one for each noise condition, were analysed resulting in a sample size of $n=85$ for first- to second-graders, $n = 90$ for third- to fourth-graders, $n = 125$ for adults and an overall sample size for all groups of $n = 300$. The correlation results are presented in Table 1.

**Table 1:** The results of the correlation between subjective listening effort scores and the experimental results on listening effort

Age groups	Correlation parameters	Correlation between ER primary task & Questionnaire score	Correlation between ER secondary task & Questionnaire score	Correlation between RT primary task & Questionnaire score	Correlation between RT secondary task & Questionnaire score
All	Spearman’s $r_s$	0.250*	0.127*	0.074	0.042
	Two-tailed significance *p*	**0.000**	**0.028**	0.200	0.470
Adults	Spearman’s $r_s$	0.409**	0.503***	0.246*	0.026
	Two-tailed significance *p*	**0.000**	**0.000**	**0.006**	0.774
1st-2nd Grade	Spearman’s $r_s$	0.300**	0.124	0.032	0.044
	Two-tailed significance *p*	**0.005**	0.259	0.771	0.687
3rd-4th Grade	Spearman’s $r_s$	0.625***	0.519***	0.560***	0.110
	Two-tailed significance *p*	**0.000**	**0.000**	**0.000**	0.303

Significant correlations are marked in bold. The number of asterisks indicate the correlation strength^46^: * - weak ($|r_s| \ge 0.10$), ** - medium ($|r_s| \ge 0.30$), or *** - strong ($|r_s| \ge 0.50$).

## Discussion

This study aimed to answer the question of how effortful listening is in classroom scenarios. To this extent, four main hypotheses were formulated. First, the objective was to verify if the dual-task paradigm could detect differeces in listening effort by checking for significant differences between single and the dual-task of the secondary task. Then, it was hypothesised that different noise conditions would affect word recognition performance as well as dual-task performance. Finally, the aim was to investigate the correlation between the listening experiment results and the subjective assessment of listening effort with the newly designed questionnaire.

In their review paper, Gagné et al.^4^ reported that listening effort is calculated as the difference in performance between the single and the dual-task conditions of the secondary task. The same analysis approach was used in the presented study. Since a difference was found for the error rate of the digit recall task between single and dual-task conditions, the presented paradigm could measure listening effort for first- to second-graders and third- to fourth-graders. This finding is in accordance with the result of Howard et al.^19^.

An additional indicator of listening effort is the comparison of performance in digit recall for different noise conditions^22^. As the differences in noise blocks in third- to fourth-graders indicated greater listening effort for R1, R2, and A2 compared to the Q condition, the present study indicates a way to measure listening effort for noise conditions compared to silence. These results are consistent with the results of the dual-task study by Howard et al.^19^ in which the age group was comparable (9- to 12-year-olds) to the one in this study (8- to 10-year-olds). However, significant differences in listening effort between the noise conditions (for different SNRs or the added room effects) were not found in third- to fourth-graders even though an increasing trend in secondary task ER was visible for more challenging noise scenarios (e.g., for A1 compared to R2 the ER increased by 0.12). As Prodi and Visentin^47^ found differences in listening effort between reverberation times (0.57 s to 0.69 s) in a study measuring response times in a speech perception task, it seems as if single task measures could be more sensitive to detect differences in listening effort between noise conditions. McGarrigle^17^ found that word recognition single tasks are more sensitive than visual dual-tasks for investigating listening effort. The presented study’s findings extend this assumption to digit recall as a secondary task since it draws from the same pool of cognitive resources as word recognition. This leads to a partial rejection of the third hypothesis because the performance in the dual-task condition did only show differences between noise and silence and not between the noise conditions, e.g. for different SNRs.

Since differences between single and dual-task performance on digit recall in the silent condition occurred in first- to second-graders, differences between the noise conditions in digit recall were expected. However, this was not the case. A possible explanation is that the secondary task was too difficult for first- to second-graders under noise. This is underlined by the guessing probability for the digit recall. Taking the probability of guessing each digit correctly after the Rencontres numbers^48^ and then calculating the expectancy value: $0*0.3667 + 1 * 0.375 + 2 * 0.1667 + 3* 0.0833 + 4 * 0 + 5 * 0.0083 = 1.000$, the expected number of correctly guessed digits for one trial is one digit. This means that on average, one digit was recalled correctly by chance corresponding to an average error rate of 80%. Considering the error rate in the digit recall for first- to second-graders, it is nearly 80% for the noise conditions. This supports the theory that some children guessed the digits under noise as the task became too difficult. A further reduction of digits to facilitate the secondary task for first- to second-graders would not be possible to mitigate the probability of guessing. This indicates that another paradigm might be needed to investigate listening effort in six- to seven-year-olds. This study is, to our knowledge, the only one investigating listening effort in children with a dual-task paradigm where the mean age of one age group was between six and seven years. Thus, these results should be considered for future dual-task research on listening effort in that age group.

For adults, no significant effect was detected for differences between single and dual-task performance in the secondary task or between noise conditions. Thus, the paradigm appeared to be not suitable for investigating listening effort in adults. This was not expected as adults had to remember seven digits (instead of five as the children), which was chosen according to the recommendation of the dual-task study by Rakerd et al.^49^. In future studies, task difficulty could be increased for adults, for example, by choosing nine instead of seven digits to be remembered to allow for the measurement of listening effort.

A limitation to the suitability of the paradigm was that no differences in response times were found for any of the age groups for the secondary task, neither for the comparison between single and dual-task nor for the comparison between noise conditions. Response time values were not analysed in the study of Howard et al.^19^, thus, no comparison of results was possible here. One possible explanation for not having found differences in the comparison of single and dual-task values of the digit recall in response times for the silent condition is that the single task digit recall was always due to time reasons in combination with the task introduction and therefore at the beginning of the experiment (as also done in^7,13,30^). The dual-task silent condition of digit recall, however, was balanced with the other noise conditions. Thus, participants always conducted the single task of digit recall at the beginning of the experiment, where higher response times were possible because the task was unknown. In future research including dual-task paradigms, the single task should be balanced with the rest of the experiment blocks. However, the latter explanation did not yield the results of the comparison between noise blocks, as those were balanced across participants.

The presented paradigm investigated not only listening effort but also the effect of close-to-real-life noise conditions on word recognition. Word recognition was inhibited by the decrease in the SNR and by adding room effects for children of all grades and adults. The room effects seemed to be roughly comparable to a decrease in the SNR by 3 dB regarding word recognition in first- to second-graders. In a study of the effect of reverberation on listening effort using a dual-task paradigm, Picou et al.^20^ reported no effect of reverberation ($T_{30}$ = 0.834 s compared to $T_{30}<$ 0.1 s) on word recognition or listening effort for children aged ten to seventeen years. The effect of the room on word recognition found in the present study could be explained by the different age groups participating (six- to ten-year-olds compared to ten- to 17-year-olds in Picou et al.^20^) which is in line with the finding that no significant differences between adults were found for anechoic effects compared to room effects in this study. Additionally, Prodi and Visentin^47^ argued that Picou et al.^20^ did not find any effect of reverberation because of their combination of unfavourable SNRs and a too small source-receiver distance. In their study, Prodi and Visentin^47^ found an effect of reverberation on speech intelligibility score in the presence of background noise when comparing 0.57 s to 0.69 s reverberation time which is in line with the results found by this study. In summary, these results manifest that it is important to consider room effects when investigating word recognition because they have an influence and better represent reality than do anechoic acoustic scenarios.

The difference in word recognition between third- to fourth-graders and adults indicates that the development in speech recognition is still ongoing after primary school (age $\geqslant$ 10 years). This is underlined by the finding that no differences in response times were found for adults in the word recognition task. However, first- to second-graders and third- to fourth-graders showed significant differences in response times for the different noise conditions indicating that more time was needed for speech processing under noise for children of this age. Considering the word recognition task’s response time as an indicator of listening effort, as suggested by previous studies^8,10,11^, the variations in response time between noise conditions for children may also reflect differences in listening effort. Additionally, the significant improvement in speech recognition in third- to fourth-graders compared to first- to second-graders at an SNR of 0 dB implies that the development of speech processing under noise is ongoing after the age of seven. This is in line with the findings of Blandy and Lutman^50^ that speech recognition under noise is worse for seven-year-olds than in adults indicating an ongoing development after the age of seven years.

To investigate the fourth hypothesis on the correlation of behavioural and subjective listening effort, children’s subjective listening effort was assessed in addition to their behavioural listening effort. The designed questionnaire led to Cronbach’s $\alpha$ values of 0.607 to 0.653. To put this into perspective, $\alpha$ values between 0.6 and 0.8 are considered acceptable^51^. Further, the comparable INCH questionnaire^52^ that assessed noise in children reached $\alpha$ values between 0.52 and 0.67. This indicates that the questionnaire was internally consistent. The results showed, as expected, that subjective perceived listening effort increased with the noise level.

The subjective assessment of listening effort was correlated to performance in the speech recognition task for all age groups. This was similarly reported by Picou et al. for 10- to 17-year-olds^20^. Furthermore, in this study, the subjective assessment of listening effort was correlated to response times in the word recognition task for third- to fourth-graders and adults which was not the case in the study by Picou et al.^20^. This occurrence could be explained by the fact that Picou et al.^20^ asked for subjective ratings of performance, ease of listening, and control, while this study participants were asked for performance, well-being and effort. Notably, the results showed a correlation between subjective and behavioural listening effort (secondary task ER) for third- to fourth-graders and adults. This is particularly interesting because a recent literature review^27^ revealed that generally poor correlations between different measures of listening effort occur. The missing correlation for first- to second-graders could be explained by the high error rates in the secondary task which could not indicate differences in behavioural listening effort. These findings support the theory by Francis and Love^9^, who stated that these three attributes (performance, well-being, and effort) describe the subjective perception of listening effort well. It is therefore recommended that future studies in different age groups also include these three questions for subjective assessment of listening effort.

Looking back at the study’s hypotheses, the following can be concluded for each hypothesis:

1. The dual-task paradigm was suited to measure listening effort by comparing single and dual-task performance in digit recall for first- to second-graders and third- to fourth-graders, but not for adults. Thus, the first hypothesis is partly accepted.
2. It was hypothesised that performance (measured as ER and RT of the secondary task and RT of the primary task) would differ significantly between noise scenarios in the dual-task condition. Results yielded significant differences in the secondary task’s ER only for third- to fourth-graders when comparing quiet and noise. However, no significant differences between noise scenarios (excluding quiet) were found, though an increase in error rate in the secondary task was noted for more complex noise scenarios. For the secondary task’s RT, no significant differences between noise scenarios occurred for any age group. Regarding the primary task’s RT, significant differences between noise scenarios were found in first- to second-graders and third- to fourth-graders, but not in adults. This leads to a partial acceptance of the second hypothesis indicating that the paradigm is only suited to assess listening effort differences between quiet and noise in third- to fourth-graders (aged eight to ten).
3. The third hypothesis assumed that the ER in the word recognition task increased with more challenging noise scenarios. Based on the present study’s results, this hypothesis is accepted for all age groups underlining the need to integrate spatial close-to-real-life noise scenarios in listening experiments to better represent real-life listening conditions.
4. A significant correlation between behavioural (secondary task’s ER) and subjective listening effort existed for third- to fourth-graders and adults. Thus, the fourth hypothesis is partly accepted. The results indicate a recommendation to use the developed questionnaire in future studies.

## Data Availability

The datasets collected and analysed during the current study are available from the corresponding author, J.S., on reasonable request. The MTB noise^34^ and the virtual room model^37^ used are freely accessible.
